# High dose proton and photon-based radiation therapy for 213 liver lesions: a multi-institutional dosimetric comparison with a clinical perspective

**DOI:** 10.1007/s11547-024-01788-w

**Published:** 2024-02-12

**Authors:** Marco Lorenzo Bonù, Luca Nicosia, Ana Turkaj, Edoardo Pastorello, Paola Vitali, Francesco Frassine, Cristian Toraci, Luigi Spiazzi, Marco Lechiara, Barbara Frittoli, Luigi Grazioli, Paolo Ghirardelli, Gianluca Costantino, Fernando Barbera, Paolo Borghetti, Luca Triggiani, Nazario Portolani, Michela Buglione, Francesco Dionisi, Irene Giacomelli, Andrea Lancia, Stefano Maria Magrini, Davide Tomasini

**Affiliations:** 1 Department of Radiation Oncology, Istituto del Radio O. Alberti, University of Brescia and Spedali Civili Hospital, Piazzale Spedali Civili 1, 25121 Brescia, Italy; 2 Department of Radiation Oncology, Ospedale Sacro Cuore Don Calabria, Negrar, Italy; 3 Proton Therapy Center, Trento, Italy; 4 Department of Medical Physics, Spedali Civili di Brescia, Brescia, Italy; 5 Department of Radiology, Spedali Civili di Brescia, Brescia, Italy; 6 Department of Radiation Oncology, Humanitas Gavazzeni Hospital, Bergamo, Italy; 7 Department of Surgery, Spedali Civili di Brescia, Brescia, Italy; 8 Radiation Oncology, Regina Elena Institute, IFO, Rome, Italy; 9 Department of Radiation Oncology, Fondazione IRCCS Policlinico San Matteo, Pavia, Italy

**Keywords:** Liver metastasis, HCC, Cholangiocarcinoma, SRT, SBRT, Proton therapy

## Abstract

**Background:**

Stereotactic radiotherapy (SRT) and Proton therapy (PT) are both options in the management of liver lesions. Limited clinical-dosimetric comparison are available. Moreover, dose-constraint routinely used in liver PT and SRT considers only the liver spared, while optimization strategies to limit the liver damaged are poorly reported.

**Methods:**

Primary endpoint was to assess and compare liver sparing of four contemporary RT techniques. Secondary endpoints were freedom from local recurrence (FFLR), overall survival (OS), acute and late toxicity. We hypothesize that Focal Liver Reaction (FLR) is determined by a similar biologic dose. FLR was delineated on follow-up MRI. Mean C.I. was computed for all the schedules used. A so-called Fall-off Volume (FOV) was defined as the area of healthy liver (liver-PTV) receiving more than the isotoxic dose. Fall-off Volume Ratio (FOVR) was defined as ratio between FOV and PTV.

**Results:**

213 lesions were identified. Mean best fitting isodose (isotoxic doses) for FLR were 18Gy, 21.5 Gy and 28.5 Gy for 3, 5 and 15 fractions. Among photons, an advantage in terms of healthy liver sparing was found for Vmat FFF with 5mm jaws (*p* = 0.013) and Cyberknife (*p* = 0.03). FOV and FOVR resulted lower for PT (*p* < 0.001). Three years FFLR resulted 83%. Classic Radiation induced liver disease (RILD, any grade) affected 2 patients.

**Conclusions:**

Cyberknife and V-MAT FFF with 5mm jaws spare more liver than V-MAT FF with 10 mm jaws. PT spare more liver compared to photons. FOV and FOVR allows a quantitative analysis of healthy tissue sparing performance showing also the quality of plan in terms of dose fall-off.

**Supplementary Information:**

The online version contains supplementary material available at 10.1007/s11547-024-01788-w.

## Introduction

Liver metastasis represent the most frequent type of liver cancer, whereas Hepatocellular carcinoma (HCC) and intrahepatic cholangiocarcinoma (ICC) are the two most common types of primary liver cancer [1].

Even if characterized by different biology and natural history, metastatic and primary liver cancer both share the possibility to be effectively managed with local therapies [2–4]. In the past, radiotherapy played an ancillary role given the impossibility to safely deliver therapeutic dose while sparing the healthy liver [5–9]. The development of Stereotactic radiotherapy (SRT) and Proton therapy (PT) has revolutionized the role of radiation oncologist in the management of liver lesions [10, 11]. Such techniques guarantee to reach ablative doses to the target while minimizing healthy liver irradiation, making radiation induced liver disease (RILD) a relative rare adverse event [12].

Photon SRT techniques, such as Volumetric modulated IMRT (V-MAT) with or without flattening filter and Robotic IMRT present an high degree of conformity to the target and different possibilities concerning motion management strategies [13–17]. PT has the theoretical advantage to exploit the unique proton beams physics proprieties, with an energy deposition at a pre-specified depth, that allows to spare beam exits and consequently reduce the radiation exposure of normal tissues [18]. A Recent meta-analysis comparing efficacy and safety of these two treatment modalities did not show differences in terms of local control and suggested a possible lower toxicity with PT [12]. Advances in systemic treatments and the consequent longer survival expected in the context of metastatic disease and HCC will require local treatment optimization in terms of efficacy and healthy liver sparing [19–21]. The aim of present study is to compare plan performance and clinical results of four advanced radiotherapy techniques performed for the treatment of HCC, ICC and liver metastasis. Particular interest will be reserved in the assessment and comparison of the four different techniques in terms of healthy liver sparing.

## Materials and methods

The present study is a multicentric, retrospective analysis run by 4 Radiation Oncology Center. The local Ethical committee approved the study. Inclusion criteria were:

- Localized HCC, ICC, or liver metastasis.
- Treatment performed with photon SRT using three techniques, robotic delivered Cyberknife® M6 Linac, V-MAT 5 mm jaws flattening filter free (V-MAT FFF 5 mm) and V-MAT 10 mm jaws with flattening filter (V-MAT FF 10 mm).
- Treatment performed with PT with active scanning technique.
- Biologic effective dose (BED) of more than 70 Gy (alpha beta ratio of 10 Gy for the tumor).
- ECOG (Eastern Cooperative Oncology Group criteria) Performance status ≤ 2
- Patients ineligible for surgery or radiofrequency ablation because of tumor site/characteristics, advanced age, comorbidity or procedure refusal
- Child–Pugh class up to B7
- All patients were staged with contrast enhanced computed tomography (CT) scan. Upper abdomen MRI or PET-CT were suggested but not mandatory.
- Have participated at follow-up following protocol indication listed in the specific section.
- Additional inclusion criteria for primary endpoint analysis were:
- Dose to 95% of PTV reached at least 95% of prescription dose.
- Available DICOM images of three months follow-up contrast enhanced Hepatobiliary phase MRI (T1-weighted fat-suppressed sequences).
- Lesions more than one centimeter from the liver surface (extremely peripherical lesion excluded).

### RT planning

Simulation, planning and delivery protocols strictly depended by RT technique used. All patients were simulated and treated in supine position. When indicated, three phasic contrast enhanced CT scan was performed. For Photon SRT, patients were treated with Cyberknife, V-MAT FFF 5 mm (TrueBeam™, Varian®) and V-MAT FF 10 mm Synergy ™, Elekta®). Simulation, planning and delivery exploited 4D-ITV delivery and tumor tracking with perilesional gold fiducial marker implant.

For PT, patients were simulated in supine position, the ABC System ™ (ELEKTA®) was exploited and deep inspiration breath hold (DIBH)-triggered planning CT scan was acquired. Patients were treated with active scanning technology that allows intensity modulated proton therapy. Pre-treatment IGRT consisted in surface guidance and portal imaging during DIBH. In supplementary Table 1s is reported the RT technique used by each center.

### Follow-up protocol and assessment of tumor response

Physical examination, blood count, liver function test, total bilirubin, INR, ALP, GGT, were performed 28–30 days after SBRT and then at each follow-up. Patients were followed every 3 months the first year after treatment, every 4 months for the second year and every 6 months thereafter. Assessment of tumor response followed RECIST criteria and was made with contrast enhanced CT scan [22] and upper abdomen contrast enhanced MRI to confirm tumor response and to perform the primary endpoint analysis. Technical aspects of MRI were represented by the use of both 1.5 T and 3 T MRI (Philips®, Siemens®). Hepatobiliary phase images of contrast-enhanced MRI were acquired 20 min after the Gd-EOB-DTPA contrast medium injection, T1-weighted fat-suppressed sequences were considered for primary endpoint purpose [23].

### Study endpoints and study methodology

Primary endpoint was to assess and compare the dosimetric healthy liver sparing performance of the four RT techniques included in the present study. We firstly standardized plans in terms of dose to the healthy liver. Secondly, we identified variables useful to quantify and compare liver sparing of RT plans [Fall-off volume (FOV) and Fall-off volume ratio (FOVR)].

**Fig. 1:**
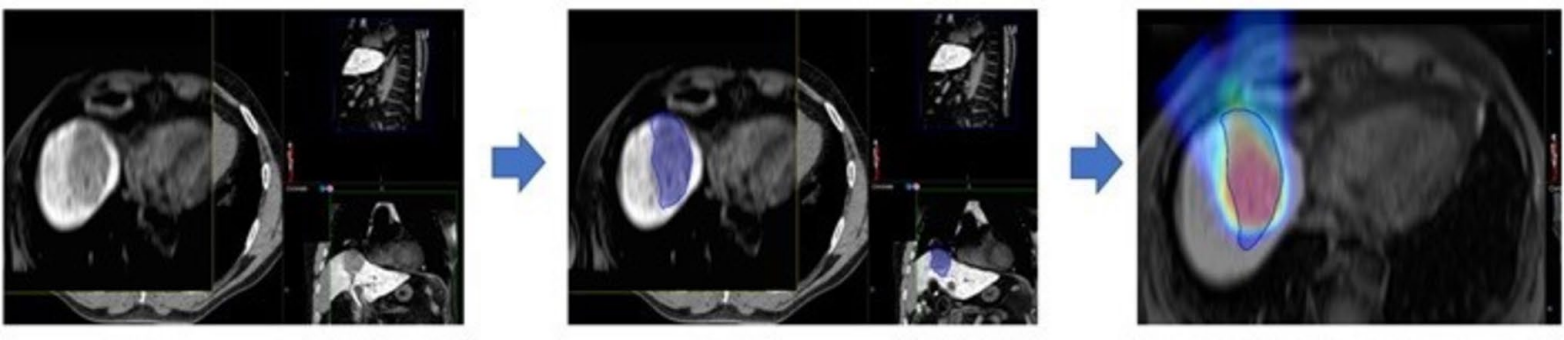
Representation of the isodose extraction process. In brief, coregistration of planning CT and 3 months follow-up MRI (hepatobiliary phase, fat-suppressed T1 weighted images), delineation on the FLR on MRI, coregistration of dose deliverd and CI extraction. Such process has been performed for all patients respecting study inclusion criteria

The methodology for the primary endpoint analysis is reported as follows:

The first step was to identify isotoxic dose resulting in liver damage for treatment using different schedules and techniques. We exploited the concept of SRT-induced area of liver damage called focal liver reaction (FLR). FLR refers to the change in imaging features of the liver parenchyma adjacent to the SRT target lesion, corresponding pathologically to veno-occlusive disease. [24] We hypothesize that such damage becomes evident at MRI for similar biologically effective doses (BED), despite the dose-fractionation used. We exploited the unique features of FLR imaging to identify BED values needed to induce FLR. We delineated on each available 3 months follow-up MRI of each patient (on hepatobiliary phase, fat suppressed T1 weighted images) the FLR. Original plans and original DICOM images of planning CT were coregistered with the corresponding follow-up MRI. Two trained radiation oncologist delineated area of FLR on each available MRI image, after the delineation a radiologist with more than 10 years of experience on liver MRI supervised and confirmed the results. To identify best fitting isodose corresponding to FLR, Conformity Index (CI) was assessed for each patient on each RT plan available and was defined as follows:

$$ CI \, = \, 2 {\text{volume }}\left( {A \, \cap \, \left( {{\text{intersect}}} \right) \, B} \right) \, / \, \left[ {{\text{volume}}\left( A \right) + {\text{ volume}}\left( B \right)} \right] $$

where “A” consists in isodose overlapping with liver and “B” the FLR. The contouring was performed on the Velocity™ software both for photons and protons treatments. A representation of the process is reported in Fig. 1. Mean CI and mean isodose related to FLR were computed for patients treated with schedules of three, five and fifteen fractions, with the objective to identifying mean isotoxic doses.

Thereafter, linear quadratic model was applied to verify radiobiologic consistency of our assumptions. Biologic effective dose was computed as follows:

$$ {\text{BED }} = \, D*[(1 \, + \, d/\left( {\alpha /\beta } \right)] $$

where “D” is the total dose, “d” is the dose per fraction; for the healthy liver α/β ratio was considered equal to 5 Gy. [25]

The second step was to identify parameters useful to compute and compare liver sparing in the standardized population.

A so-called Fall-off Volume (FOV) was conceived and defined as follows:

$$ {\text{FALL}} - {\text{OFF VOLUME }}\left( {{\text{FOV}}} \right) \, = {\text{whole liver }} - {\text{ PTV }} - \, \left( {rVx} \right){\text{ for }}3, \, 5{\text{ and }}15{\text{ fractions}}) $$

where “rVx” is the liver volume receiving less than the isotoxic dose (Gy).

**Fig. 2:**
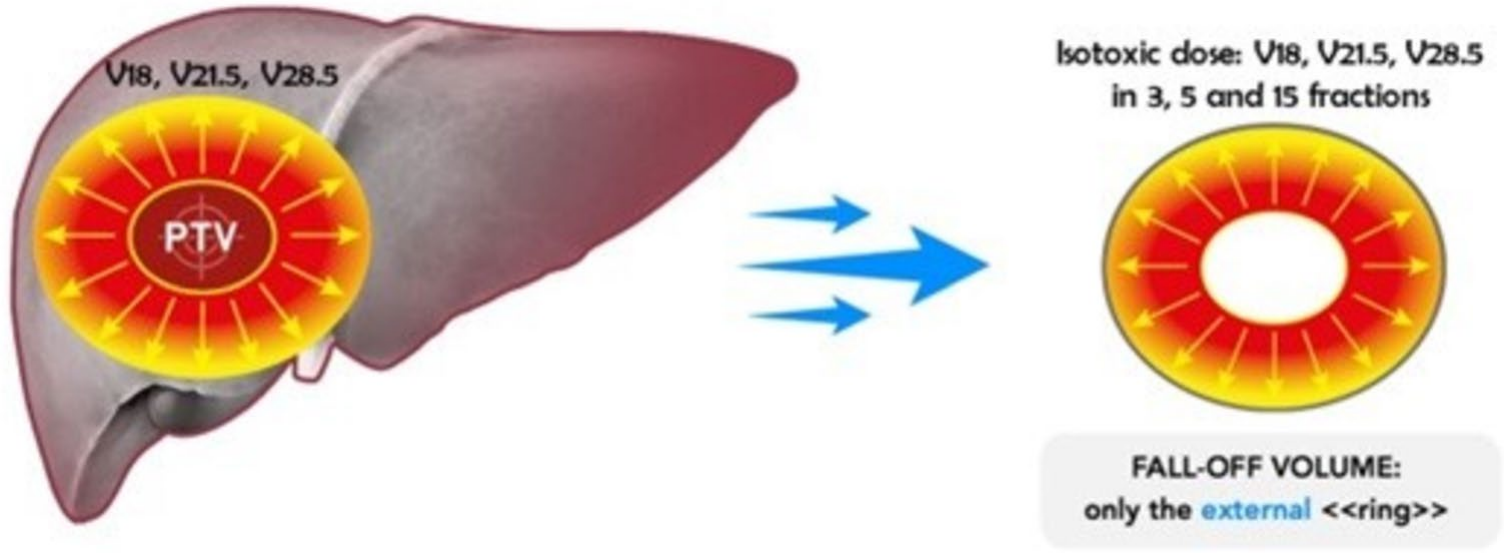
Pictorial representation of FOV computing. FOV is obtained by subtraction of whole liver volume-PTV—(rVx) for 3, 5 and 15 fractions). Where “rVx” is the liver volume receiving less than the isotoxic dose (Gy). Such volume represents the area of liver damage after SRT

The result represents the area of the healthy liver receiving at least the isotoxic dose with photon- or proton-therapy, respectively. Iconographic description of FOV is represented in Fig. 2.

Subsequently, a Fall-off volume ratio (FOVR) was conceived and defined as:

$$ {\text{FALL}} - {\text{OFF VOLUME RATIO }}\left( {{\text{FOVR}}} \right) \, = {\text{Fall}} - {\text{off Volume }}\left( {{\text{FOV}}} \right) \, /{\text{ PTV}} $$

**Fig. 3:**
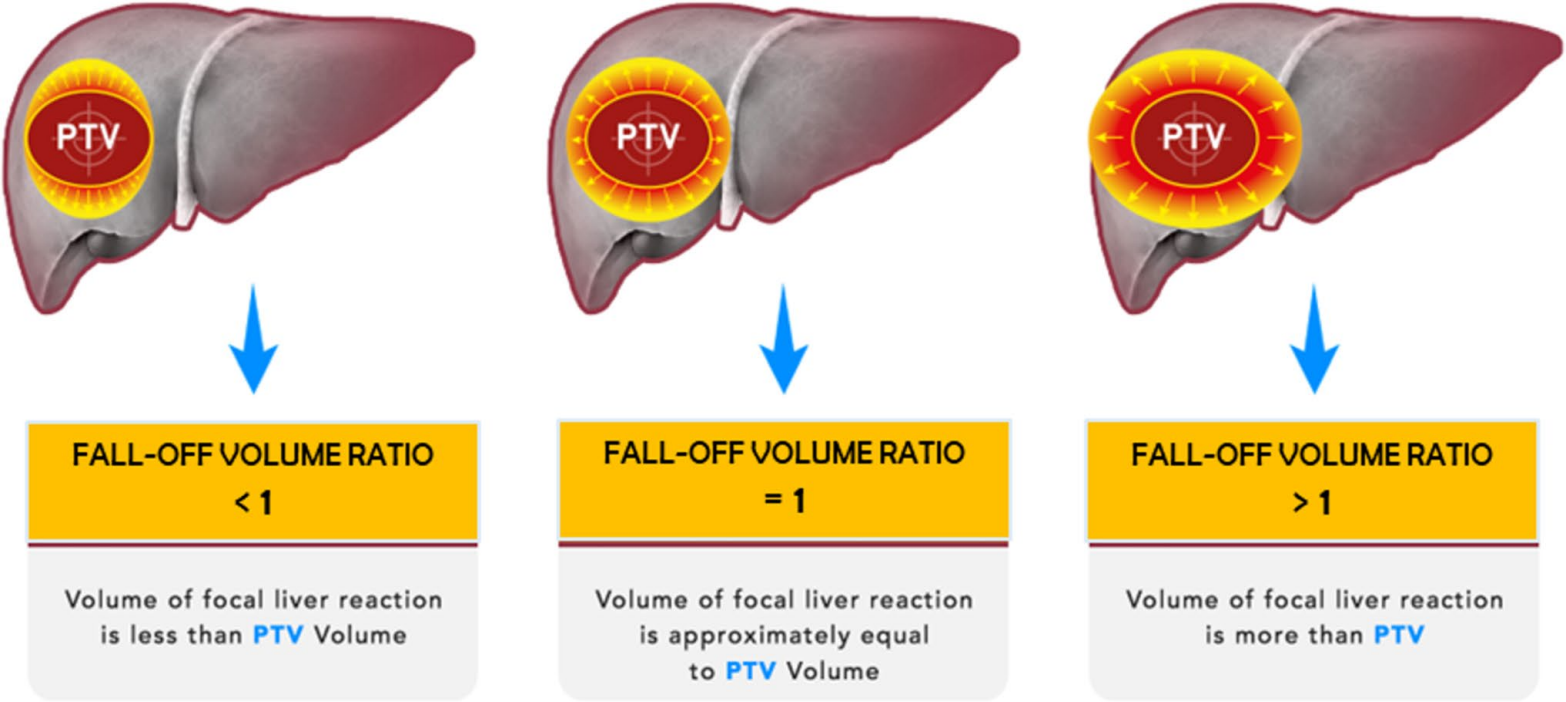
Pictorial representation of FOVR computing and interpretation of results. FOVR is obtained dividing fall-off Volume (FOV) / PTV. Such ratio represents a way to show the quantity of liver damaged in relationship to the treated PTV

FOVR is a quantitative representation reflecting the steepness of dose fall-off in relationship to PTV dimension. Therefore, FOVR can result as reported in Fig. 3.

Secondary endpoints were freedom from local recurrence (FFLR), overall survival (OS), acute and late toxic effects, assessed with the National Cancer Institute Common Terminology Criteria for Adverse Events (CTCAE), version 5.0 [26];

### Statistical analysis

For statistical analysis concerning FOV and FOVR, normality of the distributions was assessed using the Kolmogorov–Smirnov test, continuous variables were presented as medians, and min/max (in case of a skewed distribution). Comparison between FOV and FOVR between techniques were performed with the use of the Mann–Whitney and Kruskal–Wallis test; a two-sided α level of 0.05 was used for all tests.

For statistical analysis concerning secondary endpoints, the distribution of the different clinical and therapeutic features was compared with Chi square test.

Univariate analysis was performed to identify variables with a statistically significant impact on each outcome. Survival analysis was performed with the Kaplan–Meier method and the log rank test was applied to compare the effect of the individual variables on the different outcomes. A Cox proportional hazards model was planned to find independent predictors of local recurrence and survival. Univariate analysis led the selection of variables to consider as predictors. All tests were two tailed and the probability value of less than 0.05 was considered statistically significant.

Acute toxicity was defined as any adverse event occurring from the beginning of the RT up to 180 days afterward. Late toxicity was defined as any adverse event occurring at least 181 days after RT. Concerning RILD [27] this definition was used:

- CLASSIC “anicteric” radiation induced liver disease (RILD): ascites, hepatomegaly, fatigue, abdominal pain, ALP × 2 1–3 months after RT (Type 1)
- NON-CLASSIC RILD: jaundice with elevated transaminase × 5, without increase in ALP OR a decline in liver function (measured by a worsening of Child–Pugh score by 2 points or more (Type 2)

Gathered data were analyzed using SPSS® v.26.0 software (IBM®).

Moreover, given the rarity of RILD in modern series, an eventual upgrade in CHILD–PUGH class was scored after radiotherapy.

## Results

The current study was carried out according to the Declaration of Helsinki and was approved by local ethical committee. Between February 2014 and May 2021, a total of 178 patients bearing 213 hepatic lesions meeting the inclusion criteria were identified. Median age was 72 years (range 38–88 years). Majority of patients presented with Child–Pugh class A5 (87%). Hepatocellular carcinoma was the most frequent type of lesion included in the study (39%), 6% were ICC, whereas almost 55,1% of the lesions were metastasis from various kinds of primary cancers. Median lesion diameter was 2,3 cm (range 0,5–13 cm) while median liver volume was 1348 cc (range 689–3189 cc). Eligible patient’s characteristics are available in supplementary material Table 2s.

Table 1 reports treatment characteristics. Radiation therapy techniques were classified as follows: 62 lesions (29%) were treated with V-MAT FFF 5 mm, 74 (35%) with V-MAT FF 10 mm, 50 (23%) of lesions were treated with robotic SRT with Cyberknife™ and 27 (13%) with protons. The most frequent treatment schedules were 60 Gy/3 fractions (43% of the cases) for photon SRT, while proton therapy schedules delivered 58–67.5 Gy in 15 fractions (13%). Concerning target characteristics, median GTV was 6.4 cc (range 0.13–789.1 cc) and median PTV was 29.3 cc (range 4.4–1318.9 cc).

**Table 1:** Description of the series concerning technique, planning, organ motion management, RT delivery, RT schedule and target characteristics

Variable	*N*° (%)
*Technique* V-MAT FFF 5mm V-MAT FF 10mm Cyberknife Proton-therapy	62 (29%) 74 (35%) 50 (23%) 27 (13%)
*Organ motion management* 4D-ITV Active Breathing Control Tumor tracking	136 (64%) 27 (13%) 50 (23%)
*IGRT* Portal Fiducial tracking CBCT	27 (13%) 50 (23%) 136 (64%)
*Treatment schedules* 60 Gy/3 fr 48 Gy/3 fr 50 Gy/5 fr 58–67.5 Gy/15 fr Others	92 (43%) 48 (23%) 41 (19%) 27 (13%) 5 (2%)
*GTV* 0–3 cc 3,1–7 cc 7,1–20 cc > 20 cc	Median 6,4 cc (range 0,13–789,1 cc) 55 (26%) 55 (26%) 49 (23%) 54 (25%)
*PTV* 0–15 cc 15,1–30 cc 30,1–70 cc > 70 cc	Median 29,3 cc (range 4,4–1318,9 cc) 55 (26%) 52 (25%) 55 (26%) 51 (23%)

*V-MAT* Volumetric-modulated arc therapy, *FF* flattening filter technique, *FFF* flattening filter free technique, *4D ITV* 4D CT scan and definition of internal target volume technique, *IGRT* image guided radiotherapy, *GTV* gross tumor volume, *PTV* planning target volume. All data are presented as No (%) unless otherwise indicated

Table 2 describes the different radiation therapy techniques in relation to GTV and PTV dimension. Median PTV was comprised between 20 and 30 cc for the entire population treated with photons whereas median PTV resulted 242 cc for the lesions treated with PT. Dose to the 95% of the PTV reached at least 95% of the prescription dose in 155 lesions (79%). For photons, 12% of planned treatments did not respect the criteria of ICRU 83 coverage whereas this percentage rises to 78% for PT. Supplementary Table 3s describes the different radiation therapy techniques in relation to ICRU 83 coverage of the target volume. Moreover, dosimetric details of plan quality for the whole series are provided in supplementary material Table 4s.

**Table 2:** Description of GTV and PTV characteristics in relationship to RT technique

Technique	GTV (range)	PTV (range)	n
V-MAT FFF 5mm	6.65 cc (0.6–93 cc)	29 cc (12–210 cc)	62
V-MAT FF 10mm	4.23 cc (0.13–60.54 cc)	20.6 cc (4.4–140 cc)	74
Cyberknife	9.06 cc (1.3–101.5 cc)	24 cc (4.4–121 cc)	50
Proton therapy	38.2 cc (10–789 cc)	242 cc (40–1279 cc)	27

*GTV* Gross tumor volume, *PTV* planning target volume, *V-MAT* Volumetric-modulated arc therapy, *FF* flattening filter technique, *FFF* flattening filter free technique

### Primary endpoint analysis

161 patients meet the inclusion criteria to participate in primary endpoint analysis.

The 18 Gy isodose (range 16.9–19.3Gy) resulted to be the mean best fitting isodose related to FLR for three fractions schedule (with a mean CI 0.78, range 0.74–0.91). Corresponding mean values for five and fifteen fraction schedules were 21.5 Gy (range 19.7- 22.4 Gy), with a mean CI of 0.8 (range 0.76–0.93), and 28.5 Gy (range 27–29 Gy), with a mean CI of 0.74 (range 0.7–0.91). Complete results of best fitting isodose and CI extraction for each lesion treated with three, five and fifteen fractions radiotherapy schedule are presented in supplementary Table 5s, 6s and 7s.

The linear quadratic model was used to calculate the BED for 18 Gy in three fractions (39.6 Gy in 3 fractions). We derived from this value of BED the corresponding value of the isotoxic dose in 5 and 15 fractions, which resulted 21.3 Gy and 28.6 Gy for 5 and 15 fractions, respectively. Such results confirmed the results of our MRI-based biological model. To take into account impact of background liver health, we verified isotoxic dose related to liver damage in patients with healthy liver versus patients with Cirrhosis. Of 161 patients available for the analysis, 60 presented a at radiotherapy with a Cirrhosis condition. Beneath this subpopulation, fifty four presented a Child–Pugh class A5, four patients A6 and two patients B7. Concerning RT schedule in cirrhotic patients, 45 were treated with 60Gy in 3 fractions in alternate days, two patients with 48 Gy in 3 fractions in alternate days, 19 with 67.5 Gy in 15 fractions. C.I. for FLR in the whole Cirrhotic population resulted 17.3 Gy (i.e., versus 18 Gy in the whole population) for three fractions schedule (mean C.I. 0.81). Corresponding value for fifteen fractions schedule were 27.6 Gy (i.e., versus 28.5 Gy in the whole population) (mean C.I. 0.74). At statistical analysis, comparison between dose related to FLR between non-Cirrhotic and Cirrhotic population did not reach statistical significance.

Therefore, 18 Gy in three fractions, 21.5 Gy in five fractions and 28.5 Gy in 15 fractions seem to be isoeffective in causing the FLR and presence of cirrhosis seems to have a minor or no impact in FLR we proceeded in the primary endpoint analysis.

A so-called Fall-off Volume (FOV) was defined as follows:

$$ {\text{FALL}} - {\text{OFF VOLUME }}\left( {{\text{FOV}}} \right) \, = whole \, liver \, - \, PTV \, - \, \left( {rV18, \, rV21.5 \, and \, rV28.5 \, for \, 3, \, 5 \, and \, 15 \, fractions} \right) $$

where “rV” is the liver volume receiving less than 18, 21.5 and 28.5 Gy, respectively.

The result represents the area of the healthy liver receiving at least 18 Gy (3 fractions), 21.5 Gy (5 fraction) and 28.5 Gy (15 fractions) with photon- or proton-therapy, respectively.

Median FOV resulted 158 cc, 180cc, 129 cc, 62 cc in V-MAT FFF 5mm, V-MAT FF 10mm, Cyberknife and Proton plans, respectively. Difference in FOV between proton and photons (any techniques) resulted statistically significant in favor to protons (*p* < 0.001). A significantly better FOV between photons techniques was observed for V-MAT FFF 5mm and for Cyberknife treatment compared to V-MAT 10MM (p = 0.02 and *p* = 0.014 respectively).

Median FOVR was 0,57 (range 0.1–1.54) for proton plans, whereas rises up to 4.5 (range 0.93–16) for V-MAT FFF 5 mm, 5 (range 3–9.7) for Cyberknife plans, 6.2 (range 2–24.5) for V-MAT FF 10mm. Difference in FOVR between proton and photons (any techniques) resulted statistically significant (*p* < 0.001). A significantly better FOVR between photons techniques was observed for VMAT FFF 5mm and for Cyberknife treatment compared to VMAT FF 10mm (*p* = 0.013 and *p* = 0.03, respectively). No difference between VMAT FFF 5mm and Cyberknife technique was found. A comparison between each technique concerning FOV and FOVR is presented in Table 3.

**Table 3:** Representation of fall-off volume (FOV) and Fall-off volume ratio (FOVR) accordingly to RT technique. FOV is defined as the whole liver minus PTV minus area respected from the isotoxic isodose (rV18-rV21.5-rV28 for three, five, fifteen fractions, respectively), resulting the area of healthy liver out-of PTV receiving the isotoxic dose. FOVR is defined as FOV/ PTV. Only plans covered as per ICRU 83 were considered

Technique	Fall-off volume (F0V)	Fall-off volume ratio (FOVR) (FOV/PTV)	N°	*p*
V-MAT FFF 5 mm	156 cc (74–784 cc)	4.5 (0.47–26.4)	43	
V-MAT FF 10 mm	180 cc (62–489 cc)	6.2 (1–52.7)	68	
Cyberknife	130 cc (21–383 cc)	5 (2.9–22.4)	44	
Proton therapy	87 cc (51.1–236.38 cc)	0.57 (0.18–1.37)	6	< 0,001

*V-MAT* Volumetric-modulated arc therapy, *FF* flattening filter technique, *FFF* flattening filter free technique

### Secondary endpoints analysis

Chi square test showed that patients treated with proton therapy were more likely to have more locally advanced disease and larger PTV volumes in comparison with patients treated with photons (*p* < 0,001). Median FFLR was not reached in the whole series; two and three years FFLR were 83%. At univariate analysis, factors associated with worse FFLR were: proton therapy technique (versus photon technique *p* = 0.014), BED < 124 Gy (*p* = 0.004), presence of portal thrombosis (*p* = 0.003). No difference in local control emerged between patients treated with BED = 124Gy versus more than 124 Gy. In a subgroup analysis concerning BED, BED < 124 remained significantly related with poorer local control only in case of plans that meet target coverage criteria. Multivariate analysis confirmed BED < 124 Gy (OR 3.57 I.C. 1.1–11.5, *p* = 0.033) and portal vein thrombosis (OR 5.2, I.C. 1.1–24 *p* = 0.034) to be related with a poorer local control.

Median OS was 37,5 months in the whole series. Two- and three-years OS resulted in 61,4% and 52,9%. At univariate analysis, factors associated with poorer OS were female sex (p = 0.033), ECOG PS 2 (*p* < 0.001), proton technique (*p* < 0.001), GTV ≥ 9.4 cc (*p* < 0,001) and local control not achieved (p < 0.001). Multivariate analysis confirmed GTV ≥ 9.4 cc to be related with worse OS (OR 1.7, I.C. 1.05–3, *p* = 0,039).

### RILD and acute and late toxicity

Acute toxicity was assessed for the entire population. Complete acute toxicity report is described in Table 4. Moreover, a proper evaluation for RILD diagnosis was available for 106 treatments. 2 cases of type 1 RILD were described in the cohort (one in PT group and one in a pts treated with V-MAT FF 10mm, both presented a GTV more than 150 cc); no type 2 RILD was described in the study. In both two cases symptoms were mild and resolved at 6 months. In 6 cases, an upgrade in Child–Pugh classification was registered in 6 patients (from A5 to A6) in four patients treated with PT and two patients treated with photons. Late toxicity was rare: one case of G3 cholangitis not certainly related to treatment occurred.

**Table 4:** Acute toxicity, defined as adverse events occurred form the beginning of RT to 180th day after treatment

Rild	No	Yes		N Accessed
	104	2		106

Upgrade in child–pugh class	No	Yes		
	152	6		158

Other toxicity	G2	G3	G4	
Liver enzimes increased	10	0	0	152
GGT increased	19	20	0	152
Bilirubine increased	4	3	0	152
Colangitis/colestasis	0	1	0	152
Enteritis	1	0	0	152
RIB fracture	0	1	0	152

*RILD* radiation induced liver disease, *GGT* gamma glutamil transferase

## Discussion

Several dosimetric experiences demonstrated theoretically an improved plan quality with PT compared with photon SRT. The advantages were demonstrated in terms of reduced provisional dose to organs at risk. [28–35] Healthy liver sparing is routinely considered in both PT and SRT planning, using commonly published liver dose-constraints. However, the dose-constraint represented by volume respected by a certain dose (rVx) is strictly dependent by whole liver volume, and it is not a reliable parameter of dose fall-off quality [36]. Our study identified FOV and FOVR as instruments to better measure and compare plan quality in terms of healthy liver sparing. In fact, FOV is useful to quantify absolute fall-off plan quality (in *cc*), thus representing an instrument of both quality evaluation and optimization. FOVR constitutes an index of the extent of healthy liver injured by simply reporting standardized steepness of dose fall-off in relation to PTV.

Exploiting FOV and FOVR, our results shows that Cyberknife and V-MAT FFF 5mm are similar in liver sparing performance. For both, to treat a lesion it is expected to lose a volume 4–5 times bigger of the PTV itself. V-MAT-FF 10mm performed worse than other photons technique loosing approximately 6 times more healthy liver volume in relation to PTV. Despite based on limited data, PT plans seems to perform better than photons plans. Clinical consequences of such differences are not evident in our series and remain to be demonstrated.

Concerning efficacy, radiation dose remains crucial in predicting local control, with BED equal or higher to 124 Gy presenting an advantage in local control, as reported by other authors [37, 38]. Nevertheless, BED ≥ 124 Gy is not associated with an advantage in local control if plans do not meet target coverage objectives. Such result suggests that if plan does not meet ICRU 83 requirements, switching to a different schedule could be warranted. Remarkably, in our series no simultaneous integrated protection technique (SIP) was used [39, 40]. Comparison of local control and OS between techniques is biased by heterogeneity in patient characteristics. In fact, patients treated with PT had adverse features that could explain worse local control achieved and lower OS probability. Median BED in PT group was lower than 124 Gy, and an impact in local control could not be completely excluded. Remarkably, use of more biological effective schedules for PT is currently under investigation in clinical trials [41].

Our study presents some relevant limitations beside the obvious one due to its retrospective nature. A potential weakness is that the comparisons between PT and photon radiotherapy could be biased by the characteristics of PT patients, the small number of cases, and the small subgroup of PT patients that meet inclusion criteria concerning target coverage. Conversely, our results are more robust concerning comparison between different photon techniques, given the similar characteristics of patients and lesions.

Another limit is that FOV and FOVR and their implications are not applicable to lesions that arise less than 1 cm to liver surface and for FOVR, caution must be used in comparing plans with huge differences in PTV absolute volumes. Moreover, background liver health did not significantly impact on dose related to FLR, but caution must be applied in generalizing this conclusion, given the fact that the majority of cirrhotic patients in our series presented with Child–Pugh class A5 and this could explain the absence of a clear increase in radiosensitization of liver to a certain dose in comparison to healthy liver group.

Finally, the study has been conceived to present the potential role of FOV and FOVR in modern high dose liver RT. Their use, integrated with the classical dose-constraints, could contribute to refine RT planning in terms of healthy liver sparing. Whether such parameters could result in superior quality of plans is beyond the scope of this paper and remains to be demonstrated.

Despite these limitations, to our knowledge, our series try to address a critical issue poorly considered in liver high dose RT planning. In fact, taking into consideration parameters of liver damaged and not only liver spared is a new concept in liver radiotherapy, that could open new possibilities. FOV and FOVR could be integrated into modern radiotherapy planning with the aim to better measure and optimize liver sparing.

## Conclusions

Our clinical MRI-based model shows that Robotic SRT with Cyberknife and V-MAT FFF 5mm spare more liver than V-MAT with FF 10mm. Our results suggest also that PT spares more healthy liver compared to photon SRT. FOV and FOVR are new tools to measure, optimize and compare quality of healthy liver sparing also in heterogeneous clinical situations. Demonstrating the clinical consequences of the integration of FOV and FOVR in modern RT planning is beyond the scope of our series, but better healthy liver sparing thanks to cutting edge techniques and advances in RT plan optimization may open the possibility to expand SRT and PT indications also in multifocal disease and treatment of metachronous lesions.

### Supplementary Information

Below is the link to the electronic supplementary material.

Supplementary file1 (JPG 25 kb)

Supplementary file2 (DOCX 19 kb)

Supplementary file3 (DOCX 23 kb)

## Data Availability

Research data are stored in an institutional repository and will be shared upon request to the corresponding author.
