# Supplementary figures and images for: High dose proton and photon-based radiation therapy for 213 liver lesions: a multi-institutional dosimetric comparison with a clinical perspective
Source: Radiol Med. 2024 Feb 12;129(3):497–506. doi: 10.1007/s11547-024-01788-w (PMC10942931; doi:10.1007/s11547-024-01788-w)

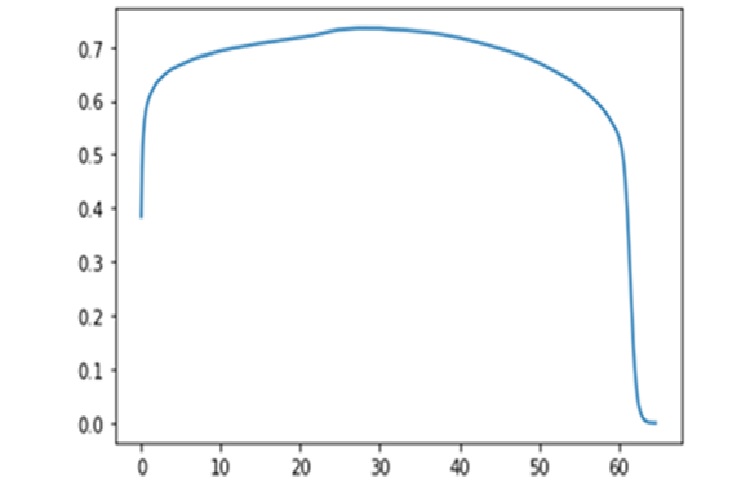

Supplement: Supplementary file 1 — Supplementary file1 (JPG 25 kb) [file 11547_2024_1788_MOESM1_ESM.jpg]
