# Supplementary material for: High dose proton and photon-based radiation therapy for 213 liver lesions: a multi-institutional dosimetric comparison with a clinical perspective
Source: Radiol Med. 2024 Feb 12;129(3):497–506. doi: 10.1007/s11547-024-01788-w (PMC10942931; doi:10.1007/s11547-024-01788-w)
Supplement: Supplementary file 2 — Supplementary file2 (DOCX 19 kb) [file 11547_2024_1788_MOESM2_ESM.docx]

Supplementary table 1s. representation of technique used by each center.

| Center | VMAT FF 10mm Jaws | VMAT FF 5mm Jaws | Cyberknife | Proton therapy |
| --- | --- | --- | --- | --- |
| Center 1 | 52 |  | 50 |  |
| Center 2 |  | 62 |  |  |
| Center 3 | 22 |  |  |  |
| Center 4 |  |  |  | 27 |

Supplementary table 2s. Patient’s and liver lesion’s characteristics at baseline.

| VARIABLE | N° (%) |
| --- | --- |
| **Age** | Median 72 y (range 38 – 88 y) |
| **Gender**  M  F | 105 (59%)  73 (41%) |
| **ECOG PS**  0  1  2 | 80 (45%)  80 (45%)  18 (10%) |
| **Child-Pugh class**  **A5**  **A6**  **B7**  **B8** | 155 (87%)  12 (7%)  7 (4%)  4 (2%) |
| **Previous medical treatment**  No  Yes | 66 (37%)  112 (63%) |
| **Previous treatment of the target lesion**  None  TACE  RFA  Alcoholization  Surgery  Radiation therapy  More than one | 149 (70%)  19 (9%)  4 (2%)  8 (4%)  19 (9%)  1 (0%)  13 (6%) |
| **Type of lesion**  HCC  ICC  Metastasis from colorectal cancer  Metastasis from lung cancer  Metastasis from breast cancer  Metastasis from pancreatic cancer  Metastasis from head and neck cancer  Metastasis from upper GI primary cancer  Metastasis from other primary cancers  Unknown | 81 (38%)  13 (6%)  40 (19%)  13 (6%)  13 (6%)  13 (6%)  2 (1%)  10 (5%)  17 (8%)  11 (5%) |
| **Target lesion maximal dimension**  0-1,5 cm  1,6 - 3 cm  > 3 cm | Median 2,3 cm (range 0,5 – 13 cm)  66 (31%)  90 (42%)  57 (27%) |
| **Total liver volume** | Median 1352 cc (range 689 – 3189 cc) |

*Legenda. ECOG PS= eastern cohoperative oncology group performace status; TACE= trans arterial chemo-embolization; RFA= radiofrequency ablation; HCC= Hepatocellular carcinoma; ICC = intra hepatic colangiocarcinoma. Note: all data are presented as No (%) unless otherwise indicated.*

Table 3s. Target coverage (following ICRU 83 recommendation) for different techniques.

| TECHNIQUE | Coverage (ICRU 83)  No yes | | total |
| --- | --- | --- | --- |
| **V-MAT FFF** | 11/62 | 51/62 | 62 |
| **V-MAT FF** | 6/74 | 68/74 | 74 |
| **Cyberknife** | 5/50 | 45/50 | 50 |
| **Proton therapy** | 21/27 | 6/27 | 27 |

*Legenda. V-MAT=* Volumetric-modulated arc therapy*; FF= flattening filter technique*; *FFF= flattening filter free technique*

Table 4s. Dosimetric parameters for the whole series

|  |  |
| --- | --- |
| **D95% PTV (%)**  < 85%  85%-95%  95% – 105%  > 105% | 16 (9%)  24 (13%)  131 (74%)  5 (3%) |
| **D2% PTV**  < 100%  100% - 120%  > 120% | 5 (3%)  148 (83%)  25 (14%) |
| **Conformity Index** | Median 0,98 (range 0,13 – 4) |
| **Homogeneity Index** | Median 1,14 (range 1,01 – 2,11) |
| **DICE** | Median 0,86 (range 0,25 – 0,99) |
