# Supplementary material for: High dose proton and photon-based radiation therapy for 213 liver lesions: a multi-institutional dosimetric comparison with a clinical perspective
Source: Radiol Med. 2024 Feb 12;129(3):497–506. doi: 10.1007/s11547-024-01788-w (PMC10942931; doi:10.1007/s11547-024-01788-w)
Supplement: Supplementary file 3 — Supplementary file3 (DOCX 23 kb) [file 11547_2024_1788_MOESM3_ESM.docx]

*Supplementary table 5s. results of best fitting isodose and conformity index extraction for each lesion treated with three fractions radiotherapy schedule.*

| PATIENT ID (3 fractions schedules, 112 lesions) | BEST FITTING ISODOSE | BEST C.I. |
| --- | --- | --- |
| SC21 | 16,9 | 0,74 |
| SC22 | 16,9 | 0,74 |
| SC23 | 16,9 | 0,74 |
| SC24 | 16,9 | 0,74 |
| SC25 | 17,1 | 0,74 |
| SC6 | 17,1 | 0,74 |
| SC7 | 17,1 | 0,74 |
| SC8 | 17,3 | 0,74 |
| SC29 | 17,3 | 0,74 |
| SC30 | 17,3 | 0,74 |
| SC31 | 17,3 | 0,74 |
| SC32 | 17,3 | 0,74 |
| SC13 | 17,3 | 0,74 |
| SC14 | 17,3 | 0,74 |
| SC15 | 17,3 | 0,74 |
| SC16 | 17,3 | 0,74 |
| SC17 | 17,4 | 0,74 |
| SC18 | 17,4 | 0,74 |
| SC19 | 17,4 | 0,74 |
| SC20 | 17,4 | 0,74 |
| SC1 | 17,4 | 0,74 |
| SC2 | 17,4 | 0,74 |
| SC3 | 17,4 | 0,74 |
| SC4 | 17,5 | 0,74 |
| SC5 | 17,5 | 0,74 |
| SC26 | 17,5 | 0,74 |
| SC27 | 17,5 | 0,74 |
| SC28 | 17,5 | 0,74 |
| SC9 | 17,5 | 0,74 |
| SC107 | 17,5 | 0,74 |
| SC108 | 17,5 | 0,74 |
| SC109 | 17,5 | 0,74 |
| SC33 | 17,5 | 0,74 |
| SC34 | 17,5 | 0,74 |
| SC49 | 17,5 | 0,74 |
| SC50 | 17,5 | 0,74 |
| SC51 | 17,5 | 0,74 |
| SC52 | 17,6 | 0,74 |
| SC53 | 17,6 | 0,74 |
| SC54 | 17,6 | 0,74 |
| SC41 | 17,6 | 0,74 |
| SC57 | 17,8 | 0,74 |
| SC58 | 17,8 | 0,74 |
| SC59 | 17,8 | 0,75 |
| SC60 | 17,8 | 0,75 |
| SC61 | 17,8 | 0,75 |
| SC62 | 17,8 | 0,75 |
| SC48 | 17,9 | 0,75 |
| SC35 | 17,9 | 0,75 |
| SC69 | 17,9 | 0,75 |
| SC70 | 17,9 | 0,75 |
| SC71 | 18 | 0,75 |
| SC72 | 18 | 0,76 |
| SC73 | 18 | 0,76 |
| SC55 | 18 | 0,76 |
| SC56 | 18 | 0,76 |
| SC42 | 18 | 0,76 |
| SC43 | 18 | 0,76 |
| SC44 | 18 | 0,76 |
| SC45 | 18 | 0,76 |
| SC46 | 18 | 0,76 |
| SC47 | 18 | 0,76 |
| SC63 | 18 | 0,76 |
| SC64 | 18,1 | 0,76 |
| SC65 | 18,1 | 0,76 |
| SC66 | 18,1 | 0,76 |
| SC67 | 18,2 | 0,76 |
| SC68 | 18,2 | 0,76 |
| SC67 | 18,2 | 0,76 |
| SC68 | 18,2 | 0,76 |
| SC36 | 18,4 | 0,79 |
| SC37 | 18,4 | 0,79 |
| SC38 | 18,4 | 0,79 |
| SC39 | 18,4 | 0,8 |
| SC40 | 18,4 | 0,8 |
| SC74 | 18,4 | 0,8 |
| SC75 | 18,4 | 0,82 |
| SC76 | 18,4 | 0,82 |
| SC77 | 18,4 | 0,82 |
| SC78 | 18,5 | 0,82 |
| SC79 | 18,5 | 0,82 |
| SC80 | 18,5 | 0,82 |
| SC81 | 18,5 | 0,82 |
| SC93 | 18,5 | 0,85 |
| SC94 | 18,5 | 0,85 |
| SC95 | 18,5 | 0,85 |
| SC96 | 18,5 | 0,85 |
| SC97 | 18,7 | 0,85 |
| SC87 | 18,7 | 0,85 |
| SC88 | 18,7 | 0,86 |
| SC89 | 18,7 | 0,86 |
| SC90 | 18,7 | 0,86 |
| SC91 | 18,7 | 0,87 |
| SC92 | 18,9 | 0,87 |
| SC82 | 18,9 | 0,87 |
| SC83 | 18,9 | 0,87 |
| SC84 | 18,9 | 0,87 |
| SC85 | 18,9 | 0,87 |
| SC86 | 18,9 | 0,87 |
| SC98 | 19,1 | 0,87 |
| SC99 | 19,2 | 0,87 |
| SC100 | 19,2 | 0,87 |
| SC101 | 19,2 | 0,88 |
| SC13 | 19,2 | 0,88 |
| SC14 | 19,2 | 0,88 |
| SC15 | 19,2 | 0,88 |
| SC16 | 19,2 | 0,88 |
| SC17 | 19,2 | 0,88 |
| SC107 | 19,3 | 0,9 |
| SC10 | 19,3 | 0,9 |
| SC11 | 19,3 | 0,9 |
| SC12 | 19.3 | 0.91 |
| **MEAN** | **18** | **0,78** |

*Supplementary table 6s. results of best fitting isodose and conformity index extraction for each lesion treated with five fractions radiotherapy schedule.*

| PATIENT ID (five fractions schedules, 43 lesions) | BEST FITTING ISODOSE | BEST C.I. |
| --- | --- | --- |
| NG21 | 19,7 | 0,76 |
| NG22 | 20 | 0,76 |
| NG23 | 20,7 | 0,76 |
| NG4 | 20,8 | 0,76 |
| NG5 | 20,9 | 0,76 |
| NG16 | 20,9 | 0,76 |
| NG17 | 20,9 | 0,76 |
| NG18 | 20,9 | 0,76 |
| NG19 | 21 | 0,76 |
| NG20 | 21 | 0,77 |
| NG11 | 21 | 0,77 |
| NG12 | 21 | 0,77 |
| SC102 | 21,2 | 0,78 |
| SC103 | 21,3 | 0,78 |
| SC104 | 21,3 | 0,78 |
| SC105 | 21,3 | 0,78 |
| SC106 | 21,4 | 0,79 |
| NG8 | 21,4 | 0,8 |
| NG9 | 21,4 | 0,8 |
| NG10 | 21,4 | 0,8 |
| NG1 | 21,4 | 0,8 |
| NG2 | 21,4 | 0,8 |
| NG3 | 21,5 | 0,81 |
| NG24 | 21,5 | 0,81 |
| NG25 | 21,6 | 0,81 |
| NG26 | 21,7 | 0,81 |
| NG27 | 21,7 | 0,81 |
| NG28 | 21,7 | 0,82 |
| NG29 | 21,7 | 0,82 |
| NG30 | 21,7 | 0,82 |
| NG41 | 21,9 | 0,83 |
| NG42 | 21,9 | 0,82 |
| NG43 | 21,9 | 0,82 |
| NG34 | 22 | 0,82 |
| NG35 | 22 | 0,83 |
| NG36 | 22 | 0,83 |
| NG37 | 22,1 | 0,83 |
| NG38 | 22,1 | 0,83 |
| NG39 | 22,3 | 0,83 |
| NG40 | 22,3 | 0,87 |
| NG31 | 22,3 | 0,87 |
| NG32 | 22,4 | 0,92 |
| NG33 | 22,4 | 0,93 |
| **MEAN** | **21,5** | **0,80** |

*Supplementary table 7s. results of best fitting isodose and conformity index extraction for each lesion treated with fifteen fractions radiotherapy schedule.*

| PATIENT ID (15 fractions schedule) | BEST FITTING ISODOSE | BEST C.I. |
| --- | --- | --- |
| PT6 | 27 | 0,7 |
| PT2 | 28,3 | 0,7 |
| PT3 | 28,7 | 0,71 |
| PT4 | 28,8 | 0,71 |
| PT5 | 29 | 0,73 |
| PT1 | 29 | 0,91 |
| **MEAN** | **28,5** | **0,74** |
